# THE LIVED EXPERIENCE OF COPD AND EMOTIONAL DISTRESS IN US VETERANS: A MIXED-METHOD STUDY

**DOI:** 10.1093/geroni/igad104.1455

**Published:** 2023-12-21

**Authors:** Patricia Bamonti, Grace Rose, Stella Park, Amy Silberbogen, Marilyn Moy, Jennifer Moye

**Affiliations:** VA Boston Healthcare System, Boston, Massachusetts, United States; VA Boston Healthcare System, Boston, Massachusetts, United States; VA Boston Healthcare System, Boston, Massachusetts, United States; VA Boston Healthcare System, Boston, Massachusetts, United States; VA Boston Healthcare System, Boston, Massachusetts, United States; VA Boston Healthcare System, Boston, Massachusetts, United States

## Abstract

Behavioral interventions targeting low physical activity (PA) and comorbid emotional distress are critically needed to address gaps in the management of chronic obstructive pulmonary disease (COPD). A mixed-methods study was conducted to guide the development of Step-Cognitive Behavioral Therapy (Step-CBT), a virtual behavioral intervention under study integrating PA promotion with CBT for those with COPD. Semi-structured interviews with U.S. Veterans with COPD at the VA Boston Healthcare System explored the lived experience of COPD and emotional distress. Participants completed the Patient Health Questionnaire-9 [PHQ-9], Beck Anxiety Inventory [BAI]), and wore an accelerometer (ActiGraph wGT3X-BT) for 14 days to measure objective PA. Thematic analysis was used to code responses and identify themes. Participants (n=22; 90.9% male, age 68.0±6.0 years; 96% White) were assessed. PHQ-9 indicated moderate depression severity (M=11.3± 5.3) and BAI scores indicated moderate anxiety severity (M=26.5±9.4). Average daily step count was (M=6,611±4,799). In response to questions eliciting participants’ experience of emotional distress, participants described Emotions, Behaviors, and Cognitions associated with distress that interacted with COPD and PA (e.g., “I’ve gotta catch my breath where before I could just do up the yard with no problem… so that gets me depressed”). When asked about engaging in daily PA in the context of emotional distress, three themes emerged: Emotion Regulation and Coping (e.g., healthy behavioral coping, maladaptive coping, etc.), Factors that Motivate (i.e., routine/schedule, etc.), and Social Context. Findings illuminate linkages between emotional distress and PA that will guide the creation of vignette-based treatment modules in the development of Step-CBT.

